# Prognostic costimulatory molecule-related signature risk model correlates with immunotherapy response in colon cancer

**DOI:** 10.1038/s41598-023-27826-7

**Published:** 2023-01-16

**Authors:** Wanze Huang, Duntao Su, Xin Liao, Tongtong Yang, Yan Lu, Zhejia Zhang

**Affiliations:** 1 Department of Breast and Thyroid, Xiangya Boai Rehabilitation Hospital, 168 Wanjiali North Road, Changsha, 410100 China; 2 Department of General Surgery, Xiangya Hospital, Central South University, No. 87 Xiangya Road, Changsha, 410008 China; 3 Department of Cardiac Macrovascular Surgery, Yueyang Central Hospital, 39 Dongmaoling Road, Yueyang, 410000 China; 4 Hunan Sany Industrial Vocational and Technical College, Changsha, China

**Keywords:** Tumour immunology, Colorectal cancer

## Abstract

Costimulatory molecules can promote the activation and proliferation of T cells and play an essential role in immunotherapy. However, their role in the prognosis of colon adenocarcinoma remains elusive. In this study, the expression data of costimulatory molecules and clinicopathological information of 429 patients with colon adenocarcinoma were obtained from The Cancer Genome Atlas database. The patients were divided into training and verification cohorts. Correlation, Cox regression, and Lasso regression analyses were performed to identify costimulatory molecules related to prognosis. After mentioning the construction of the risk mode, a nomogram integrating the clinical characteristics and risk scores of patients was constructed to predict prognosis. Eventually, three prognostic costimulatory molecules were identified and used for constructing a risk model. High expression of these three molecules indicated a poor prognosis. The predictive accuracy of the risk model was verified in the GSE17536 dataset. Subsequently, multivariate regression analysis showed that the signature based on the three costimulatory molecules was an independent risk factor in the training cohort (HR = 2.12; 95% CI = 1.26, 3.56). Based on the risk model and clinicopathological data, the AUC values for predicting the 1-, 3-, and 5-year survival probability of patients with colon adenocarcinoma were 0.77, 0.77, and 0.71, respectively. To the best of our knowledge, this study is the first to report a risk signature constructed based on the costimulatory molecules TNFRSF10c, TNFRSF13c, and TNFRSF11a. This risk signature can serve as a prognostic biomarker for colon adenocarcinoma and is related to the immunotherapeutic response of patients.

## Introduction

According to recent statistics, colorectal cancer is the third most prevalent malignant tumor worldwide, with the second highest mortality rate^1,2^. The most common pathological subtype of colorectal cancer is colon adenocarcinoma (COAD). Colon cancer is often occult, and most patients present with a change in defecation habits^3^. The overall prognosis of early-stage colon cancer is good, with a 5-year survival rate of 90%. However, many patients are diagnosed at an advanced stage, and their 5-year survival rate is approximately 14%^4^. At present, rectal cancer is mainly treated with surgery, neoadjuvant radiotherapy, neoadjuvant chemotherapy, targeted therapy, and immunotherapy. Despite breakthroughs in targeted therapy and immunotherapy in the past decade, the prognosis of patients with advanced colon cancer remains poor^5^. Therefore, identifying a new prognostic index for colon cancer is necessary for improving risk stratification, predicting survival and immunotherapy response, developing individualized treatment strategies, and improving the prognosis.

In recent years, immunotherapy has emerged as an effective treatment for malignant tumors. It can activate the immune system to attack and kill tumor cells^6^. Several recent studies have reported that costimulatory molecules can promote the activation, proliferation, and survival of T cells and regulate the secretion of cytokines from T cells. Additionally, they can regulate the response of the immune system to tumors. The number of tumor antigen-specific T cells can be increased by manipulating costimulatory molecules, leading to inhibition of tumor growth and elimination of tumor cells. This phenomenon provides novel insights into developing immunotherapy for malignant tumors^7^. Costimulatory molecules are mainly divided into two categories: B7-CD28 family and tumor necrosis factor (TNF) receptor superfamily. The members of these families are promising immunotherapeutic targets^8^. The B7-CD28 family comprises 13 costimulatory molecules^9^, and the TNF family comprises the TNF ligand superfamily (TNFSF) and the TNF receptor superfamily (TNFRSF), with a total of 48 costimulatory molecules. Many receptor–ligand pairs have been identified as positive regulators of T cells^10^. However, the role of costimulatory molecules in the treatment and prognosis of colon cancer remains unclear in clinical settings.

In this study, we systematically examined the relationship between the expression of prognostic costimulatory molecules and the clinical characteristics and immune microenvironment of patients with colon cancer. In addition, we constructed a risk model based on costimulatory molecules differentially expressed between patients with cancer and healthy individuals. According to the median risk score, patients were divided into low- and high-risk groups. An external verification dataset was used to verify the risk model, and the results indicated good predictive efficiency. Subsequently, a nomogram integrating clinical characteristics and risk scores was constructed to predict the prognosis of patients with colon cancer. Figure S1 illustrates the detailed study protocol. The novel risk model developed in this study can predict the prognosis and immunotherapy response of patients with colon cancer.

## Materials and methods

### Datasets and clinical data

Data on costimulatory molecules and matched clinical information of patients with COAD in The Cancer Genome Atlas (TCGA-COAD) dataset were extracted using the University of California Santa Cru (UCSC) Xena browser (TCGA database version: Data Release 31.0, October 29, 2021). Our inclusion criteria were patients with complete clinical information and survival information were included, whereas those with exclusion criteria were incomplete information were excluded. The GSE17536 and GSE78220 dataset from the Gene Expression Omnibus (GEO) database (https://www.ncbi.nlm.nih.gov/go/) was used as the validation cohort^11^.

### Identification of costimulatory molecules

Based on previous studies^12–15^, a total of 59 costimulatory molecules were screened, and their expression was compared between COAD and normal tissues. Based on the expression of costimulatory molecules, correlation analysis was performed to discover interrelationships. STRING (https://string-db.org/) was used to analyze the protein–protein interaction (PPI).

### Development and validation of a prognostic costimulatory-molecule-based signature

The TCGA-COAD dataset was used as the training cohort to construct a prognostic costimulatory-molecule-related signature, whereas the GES17536 dataset was used as the validation cohort to verify the predictive efficiency of the signature. Univariate and multivariate Cox regression analyses were performed to identify prognostic costimulatory molecules. The R package “survival” was used for univariate and multivariate Cox regression analyses^16^. Subsequently, the least absolute shrinkage and selection operator (Lasso) regression analysis was used to identify significant prognostic costimulatory molecules. The risk score was calculated as follows = expression of (costimulatory molecule 1) × (β1 of costimulatory molecule 1) + expression of (costimulatory molecule 2) × (β2 of costimulatory molecule 2) + … expression of (costimulatory molecule n) × (βn of costimulatory molecule n)^17^. Based on the median risk score, both training and validation cohorts were divided into low- and high-risk groups. Survival analysis was performed and receiver operating characteristic (ROC) curves were plotted to verify the predictive value of the prognostic signature. In addition, the clinicopathological information of patients was integrated with the risk scores in multivariate Cox regression analysis to verify the predictive value of the signature. Finally, a nomogram was constructed to predict the prognosis of patients. The “survminer” R package was used to compare overall survival (OS) between the low- and high-risk groups^18^. To investigate the predictive ability of the prognostic signature over time, the “TimeROC” R package was used to plot ROC curves^19^.

### Immune analysis

ImmuCellAI (http://bioinfo.life.hust.edu.cn/ImmuCellAI#!/) was used to predict immune checkpoint blockade (ICB) to assess immunotherapy response. In addition, the ESTIMATE algorithm^20^ was used to calculate tumor purity and the proportion of infiltrating stromal/immune cells in the high- and low-risk groups. Furthermore, HLA-related genes were identified, and their expression was compared between the low- and high-risk groups. HPA (https://www.proteinatlas.org/) was used to compare the protein expression levels between COAD and normal tissues.

### Gene set enrichment and functional enrichment analyses

Kyoto Encyclopedia of Genes and Genomes (KEGG) (https://metascape.org/gp/index.html#/main/step1) and Gene Ontology (GO) (https://proteomaps.net/) were used for pathway enrichment and functional annotation analyses, respectively.

### Construction of a nomogram

A nomogram was constructed based on the clinicopathological information and risk scores of patients. The “rms” R package^21^ was used to develop the nomogram for predicting the 1-, 3-, and 5-year survival probability of patients with COAD. ROC curves were plotted to evaluate the predictive accuracy of the prognostic signature. An alluvial plot was constructed to determine the outcome of patients with different clinical and pathological characteristics. Finally, decision curve analysis (DCA) was performed to verify the clinical significance of the signature. DCA curves were plotted using the “rmda” R package^22^.

### Validation via quantitative reverse transcription PCR

Two human colon cancer cell lines (HCT-116 and DCD-1) and a human normal colon epithelial cell line (FHC) were cultured in a complete medium supplemented with 10% fetal bovine serum (Gibco, Carlsbad, USA) and RPMI1640 (Gibco) or DMEM (Gibco) supplemented with 100-U/mL penicillin (HyClone) and 100-mg/mL streptomycin. The cells were cultured at 37 °C with 5% CO_2_. Total RNA was extracted from these cells and reverse transcribed (100 ng) to synthesize cDNA according to the manufacturer’s instructions. Subsequently, quantitative reverse transcription polymerase chain reaction (qRT-PCR) was performed using TBGreen Premix Ex TaqTMII (Cat # RR047A-5, TaKaRa, Japan). Primer sequences for costimulatory-molecule-mRNAs are listed in Supplementary Table S2. All experiments were performed in triplicate.

### Statistical analysis

The R software (version 4.0.1) was used for all statistical analyses. One-way analysis of variance (ANOVA) and nonparametric tests were performed as appropriate. A *P* value of < 0.05 was considered statistically significant.

### Ethics approval and consent to participate

This study was approved and agreed upon by the Ethics Committee of Xiangya Hospital of Central South University, and all patients participating in the study provided written informed consent.

## Results

### Differential expression and genetic modifications of costimulatory molecules between normal and COAD tissues

**Figure 1:**
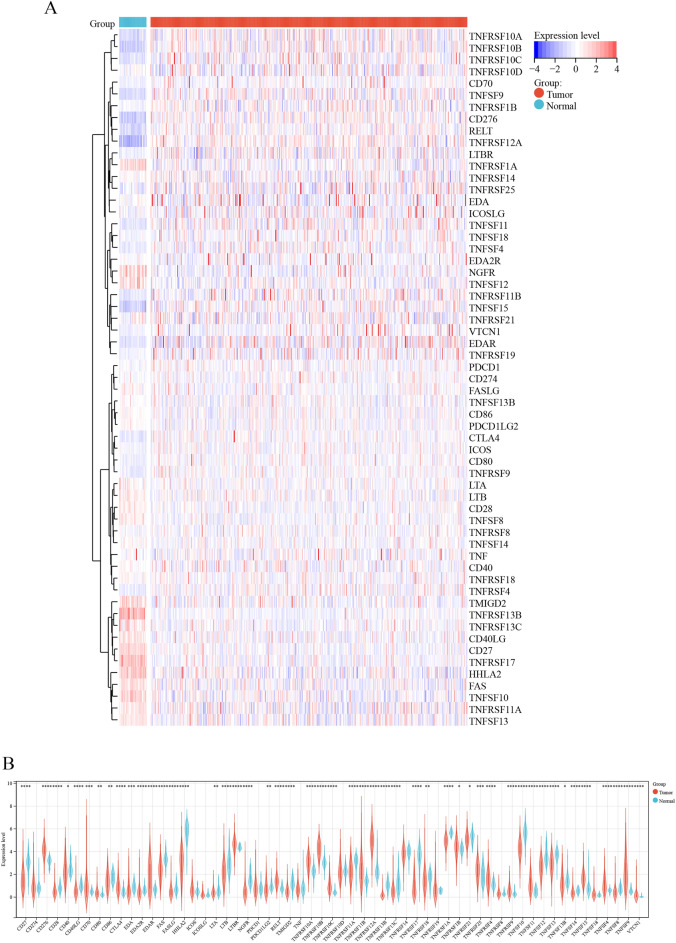
Differential expression and genetic modifications of costimulatory molecules between normal and COAD tissues. (**A**) The heatmap of 49 costimulatory molecules in normal and COAD tissues. (**B**) The expression of 49 costimulatory molecules in normal and COAD tissues.

**Figure 2:**
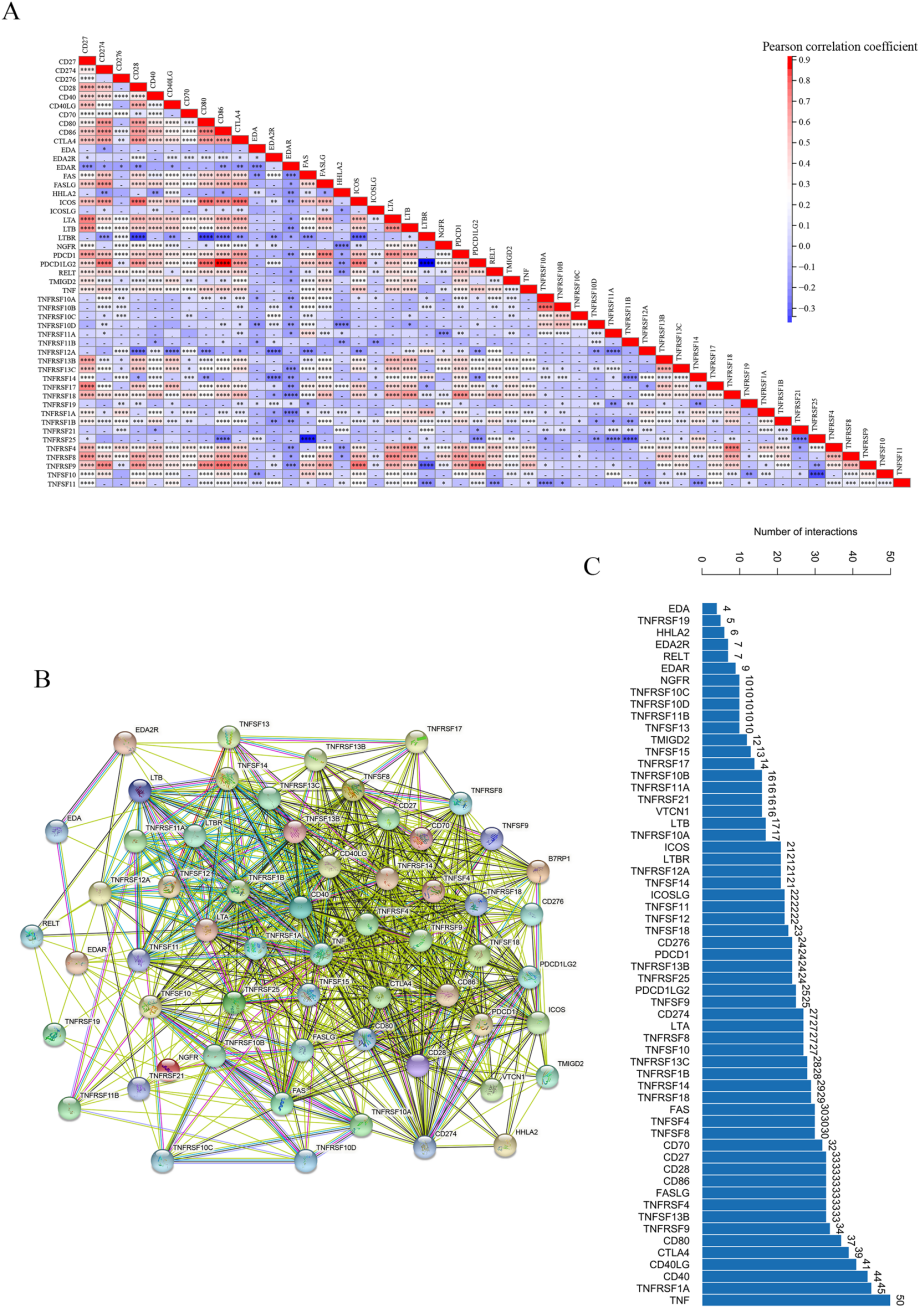
The relationship in 49 costimulatory molecules. (**A**) The heatmap of correlation among 49 co-stimulatory molecules. (**B**,**C**) The protein interaction network among 49 co-stimulatory molecules.

After excluding TNFRSF6B, which had low expression in tumor and normal tissues, 59 costimulatory molecules were selected from the TCGA-COAD dataset. These molecules included 13 costimulatory molecules from the B7-CD28 family and 46 costimulatory molecules from the TNFRSF family. The expression levels of these 59 costimulatory molecules were compared between 429 COAD and 37 normal tissues. The expression of 49 costimulatory molecules was found to be significantly different between COAD and normal tissues *(P* < 0.05). A heat map and box plot were constructed to visualize the differential expression of these costimulatory molecules between normal and COAD tissues (Fig. 1). Of the 49 costimulatory molecules, 21 molecules (VTCN1) were upregulated and 28 molecules (TNFSF8) were downregulated in tumor tissues. Figure 2A,B demonstrate the relationship among these co-stimulatory molecules. LTBR and PDCD1LG2 had the strongest negative correlation, whereas CD86 and PDCD1LG2 had the strongest positive correlation. A strong correlation was observed among all costimulatory molecules. The histogram demonstrated that TNF, TNFRSF1A, and CD40 were the most interactive proteins (Fig. 2C).

### Pathway and functional enrichment analyses

KEGG pathway enrichment and GO functional analyses were performed to examine the potential biological mechanisms associated with the costimulatory molecules in COAD and normal tissues (Figure S2). The dominant five GO terms included cytokine–cytokine receptor interaction, regulation of lymphocyte proliferation, the tumor necrosis factor-mediated signaling pathway, TNF receptor superfamily (TNFSF) mediators, and the intestinal immune network for IgA production.

### Establishment and verification of a risk signature related to costimulatory molecules

**Figure 3:**
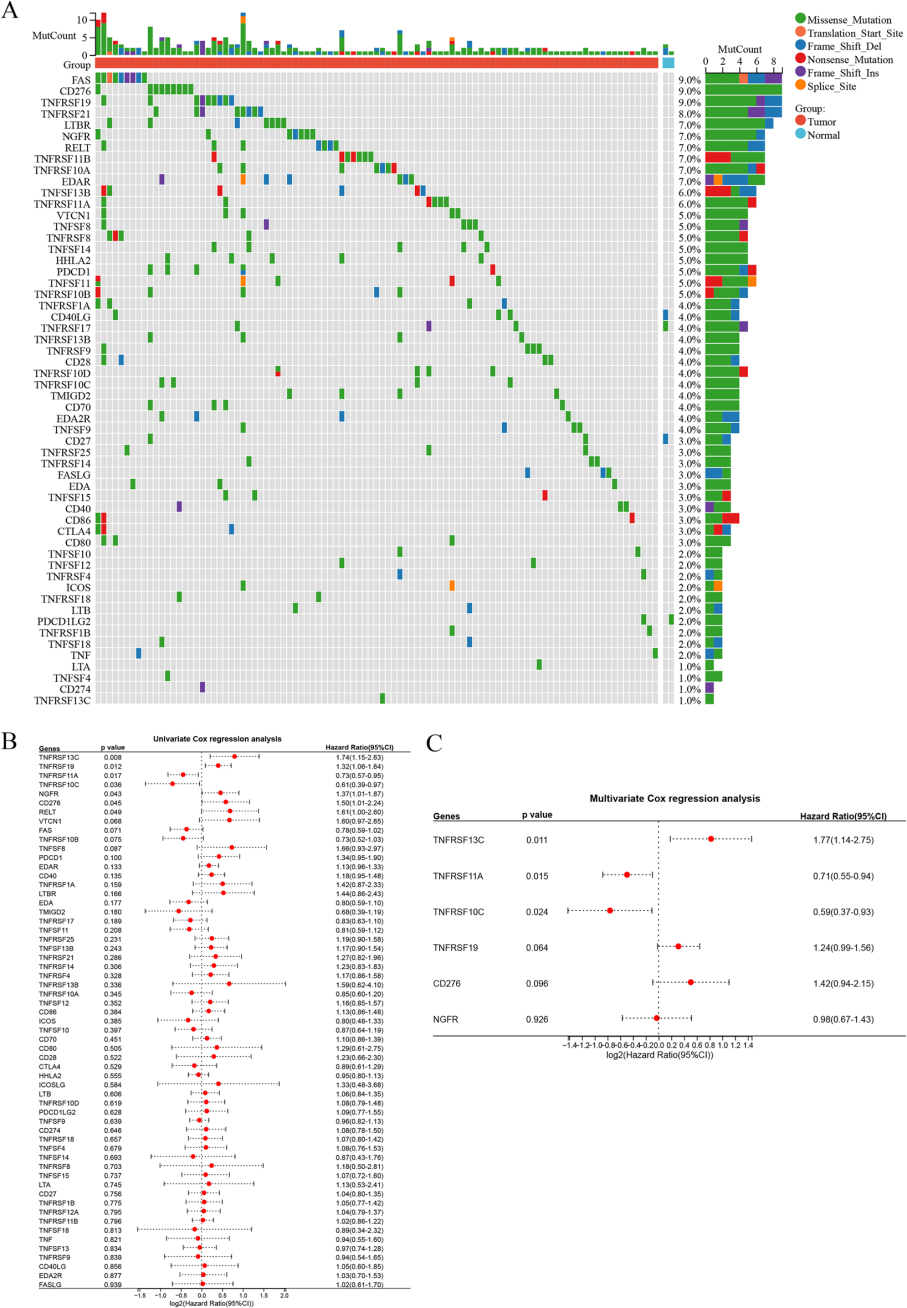
The mutation map and univariate and multivariate Cox regression analyses in costimulatory molecules. (**A**) The mutation map among costimulatory molecules. (**B**) The univariate Cox regression analyses in costimulatory molecules. (**C**) The multivariate Cox regression analyses in costimulatory molecules.

**Figure 4:**
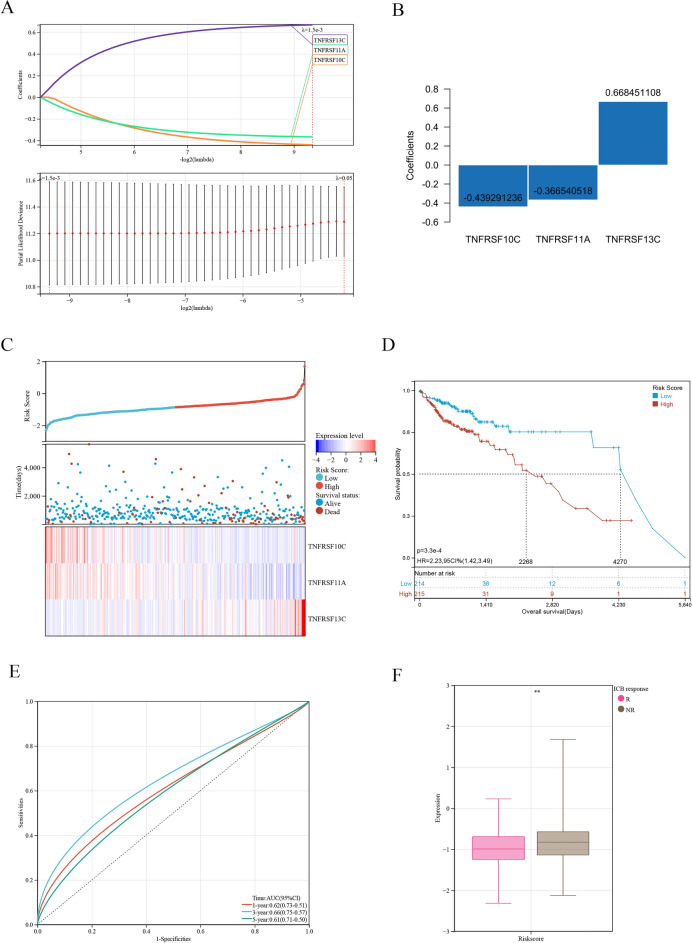
Establishment and verification of a risk signature related to costimulatory molecules (**A**, **B**) The Lasso–Cox regression analysis in costimulatory molecules. (**C**) Distribution of risk scores and overall survival status in the training cohort. (**D**) Kaplan–Meier curves for the overall survival of patients in the high- and low-risk groups in the training cohort. (**E**) The time-dependent ROC curves supporting prognostic accuracy of the risk score in the training cohort. (**F**) The ICB response of the risk score in the training cohort.

**Figure 5:**
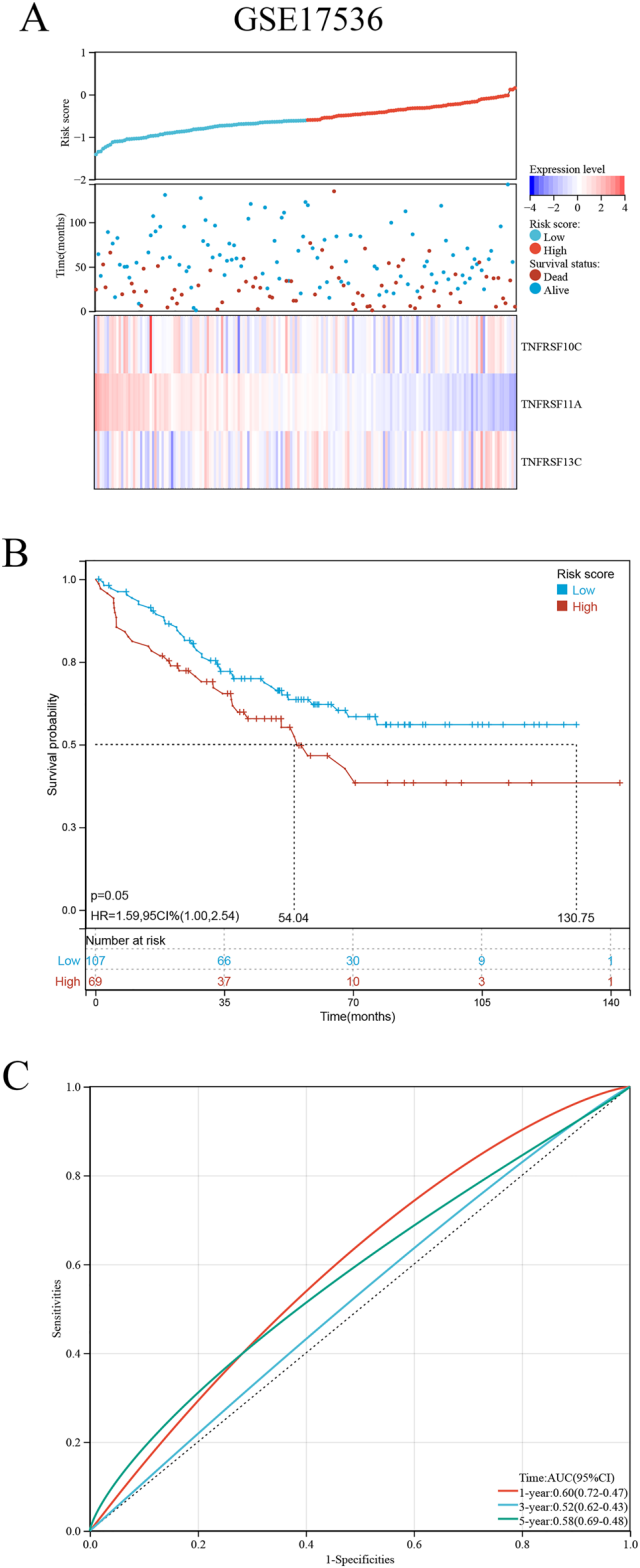
Prognostic analysis of costimulatory molecules signature in validation cohorts. (**A**) Distribution of risk scores and overall survival status in the validation cohort. (**B**) Kaplan–Meier curves for the overall survival of patients in the high- and low-risk groups in the validation cohort. (**C**) The time-dependent ROC curves supporting prognostic accuracy of the risk score in the validation cohort.

Mutations in costimulatory molecules were found in most samples (Fig. 3A), with FAS having the highest mutation rate of 9.0%. Costimulatory molecules associated with the prognosis of COAD were identified via univariate and multivariate Cox regression analyses (Fig. 3B,C). The results of multivariate Cox regression analysis were visualized on a forest plot, which demonstrated significant in the prognosis of 6 prognostic costimulatory molecules in COAD tissues. Of the 59 costimulatory molecules, 3 molecules were identified to be significantly associated with prognosis via Lasso–Cox regression analysis. These 3 molecules were used to construct a risk model (Fig. 4A). Additionally, the results of Lasso–Cox regression analysis were used to calculate the risk score of each sample. The detailed results of univariate and multivariate Cox regression analysis are shown in Table S1. Based on the λ value of 0.05, the 3 costimulatory molecules were used to build a risk model (Fig. 4B), and the risk score was calculated as follows: (TNFRSF10C) * (− 0.439291236) + (TNFRSF11A) * (− 0.366540518) + (TNFRSF13C) * (0.668451108). Subsequently, the median risk score was used as the cut-off value to divide all tumor samples into high- and low-risk groups (Fig. 4C). The higher the risk score, the shorter the survival time. As shown in the K–M curve in Fig. 4D, the prognosis of patients with higher risk scores was worse than that of patients with lower risk scores. The clinicopathological characteristics of patients in the high- and low-risk groups in the TCGA-COAD dataset are shown in Table 1. The ROC curve showed that the risk score exhibited good predictive performance, and the area under the curve (AUC) values for predicting 1-, 3-, and 5-year survival were 0.62, 0.66, and 0.61, respectively (Fig. 4E). Patients in the low-risk group responded better to immune checkpoint inhibitor (ICI)-based therapy, suggesting that patients with low risk scores are more sensitive to immunotherapy (Fig. 4F). The risk model was verified in an external validation cohort (GSE17536 dataset) using the same methods as described above (Fig. 5A). The results were consistent. The higher the risk score, the worse the prognosis (Fig. 5B). The risk score exhibited good predictive performance, and the AUC values for predicting 1-, 3-, and 5-year survival were 0.60, 0.52, and 0.58, respectively (Fig. 5C).

**Table 1:** Associations between the signature and patient characteristics in training and validation cohort.

Characteristics	TCGA-COAD				GSE17536			
	High risk (N = 214)	Low risk (N = 215)	Total (N = 429)	*P*	High risk (N = 88)	Low risk (N = 89)	Total (N = 177)	*P*
Age								
Mean ± SD	66.08 ± 12.70	67.31 ± 12.84	66.70 ± 12.77		65.64 ± 13.31	65.33 ± 12.92	65.48 ± 13.08	
Median[min–max]	68.00[31.00,90.00]	69.00[34.00,90.00]	69.00[31.00,90.00]		66.50[26.00,92.00]	66.00[30.00,89.00]	66.00[26.00,92.00]	
Gender				0.31				0.94
Female	95 (22.14%)	107 (24.94%)	202 (47.09%)		41 (23.16%)	40 (22.60%)	81 (45.76%)	
Male	119 (27.74%)	108 (25.17%)	227 (52.91%)		47 (26.55%)	49 (27.68%)	96 (54.24%)	
T stage				0.26				
T1	2 (0.47%)	7 (1.63%)	9 (2.10%)					
T2	40 (9.32%)	35 (8.16%)	75 (17.48%)					
T3	145 (33.80%)	152 (35.43%)	297 (69.23%)					
T4	27 (6.29%)	21 (4.90%)	48 (11.19%)					
N stage				< 0.01				
N0	103 (24.01%)	150 (34.97%)	253 (58.97%)					
N1	55 (12.82%)	44 (10.26%)	99(23.08%)					
N2	56 (13.05%)	21 (4.90%)	77 (17.95%)					
M stage				0.13				
M0	177 (41.26%)	190 (44.29%)	367 (85.55%)					
M1	37 (8.62%)	25 (5.83%)	62 (14.45%)					
Tumor stage				< 0.01				
Stage I	35 (8.16%)	39 (9.09%)	74 (17.25%)					
Stage II	64 (14.92%)	106 (24.71%)	170 (39.63%)					
Stage III	78 (18.18%)	45 (10.49%)	123 (28.67%)					
Stage IV	37 (8.62%)	25 (5.83%)	62 (14.45%)

### Risk model based on immune cell infiltration and clinicopathological factors

**Figure 6:**
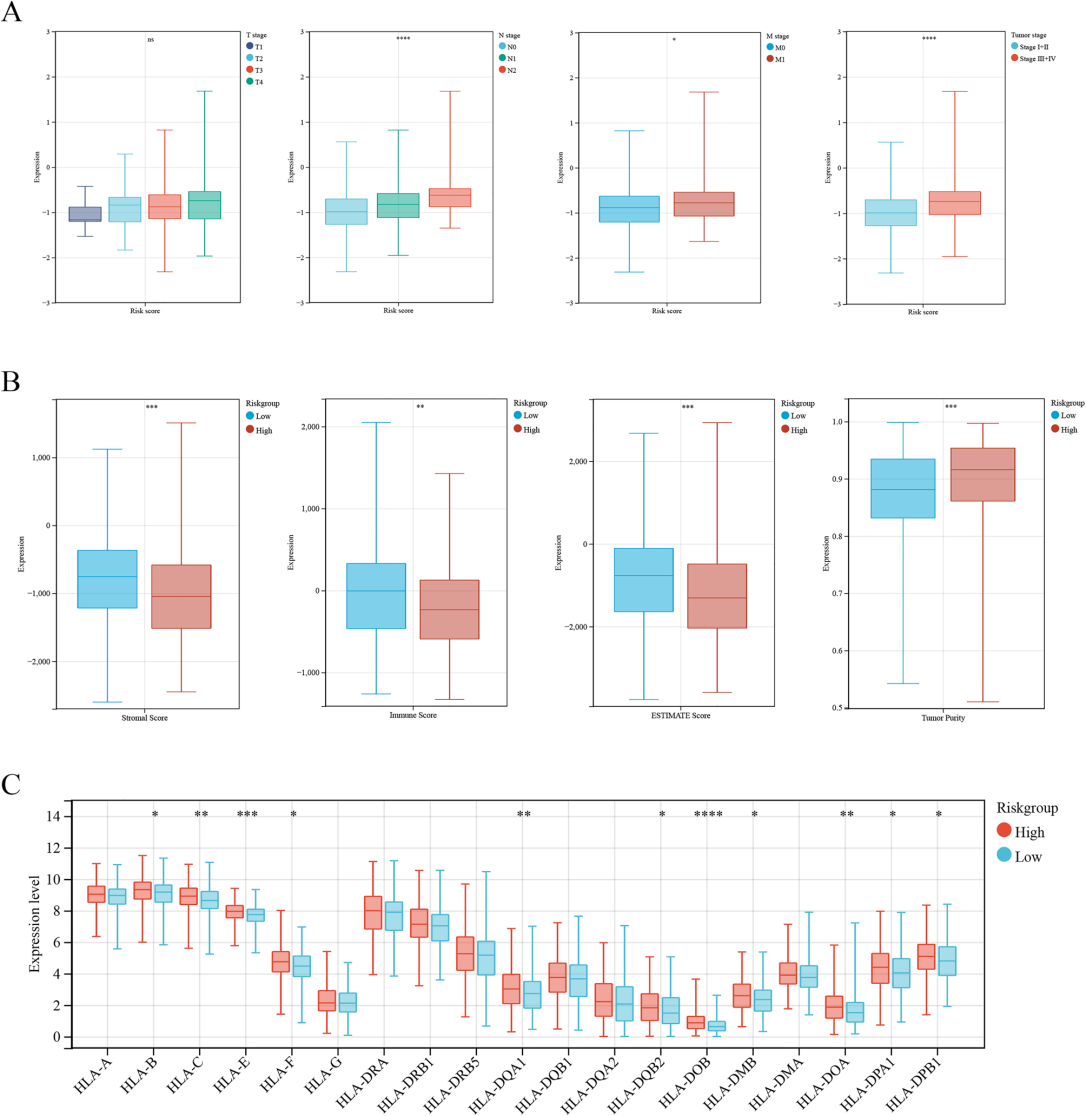
Risk model based on immune cell infiltration and clinicopathological factors. (**A**) The T stage, N stage, M stage, and tumor stage of patients between the low- and high-risk groups. (**B**) The immune, stromal, tumor purity and ESTIMATE scores between the low- and high-risk group. (**C**) The leukocyte antigens (HLAs) between the low- and high-risk group.

Significant differences were observed in the T stage, N stage, M stage, and tumor stage of patients between the low- and high-risk groups (Fig. 6A). The immune, stromal, and ESTIMATE scores were lower and tumor purity was significantly higher in the high-risk group (Fig. 6B). Among 19 human leukocyte antigens (HLAs), HLA-DPA1 and HLA-DQB1 were significantly enriched in the high-risk group (Fig. 6C).

### Validation of risk models and in vitro experiments

**Figure 7:**
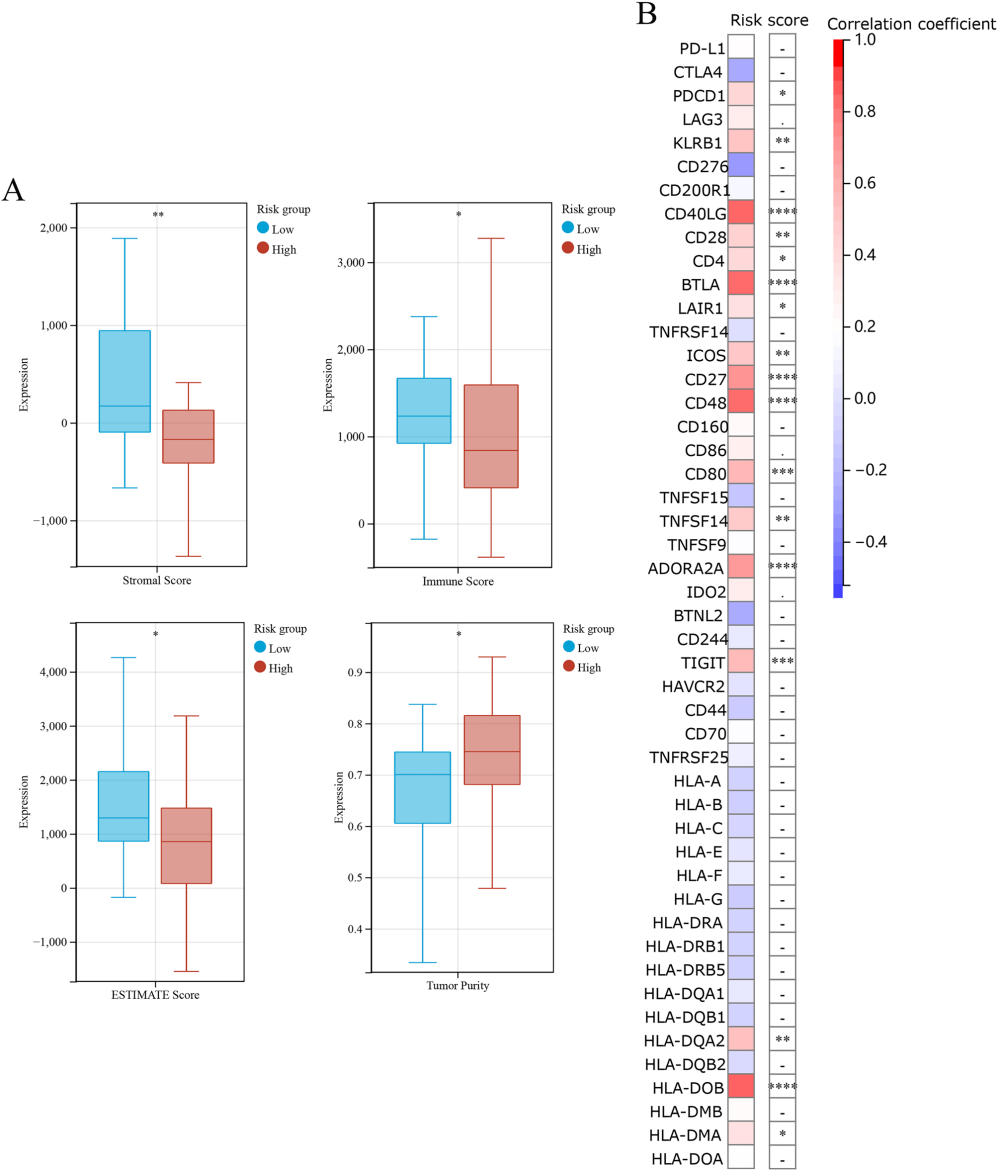
Validation of risk models (**A**) The immune, stromal, tumor purity and ESTIMATE scores between the low- and high-risk melanoma group (GSE78220). (**B**) The heat map of immune checkpoints, HLA and immune receptors in melanoma cohort.

**Figure 8:**
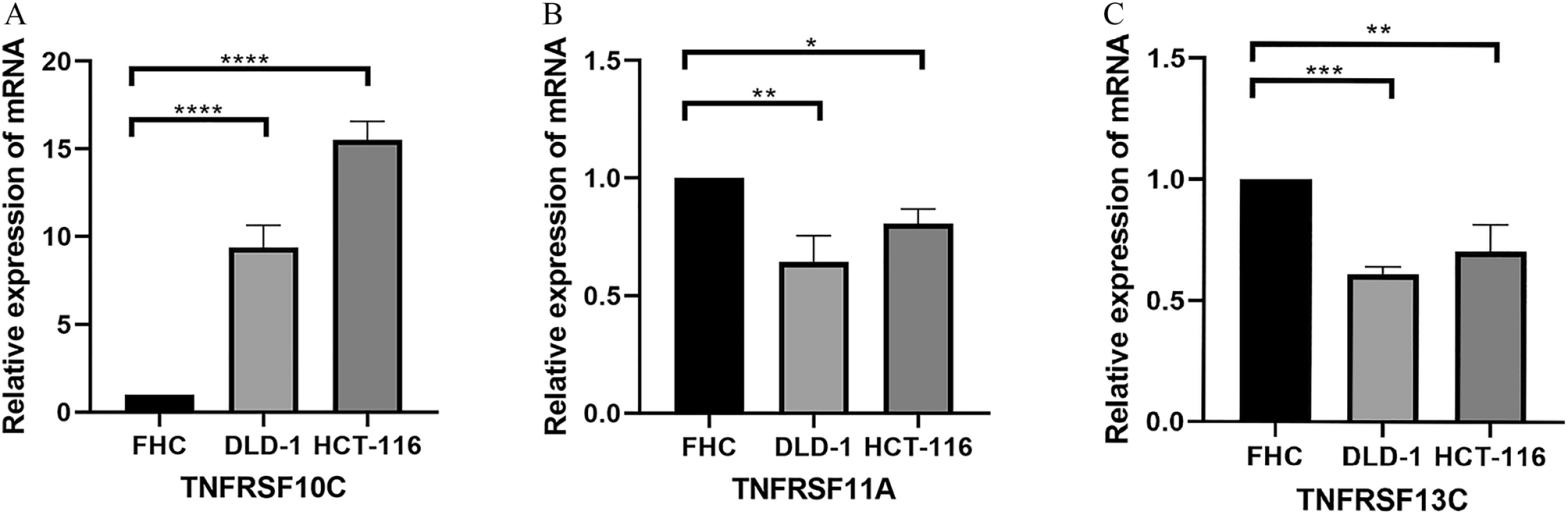
Validation in vitro experiments (**A**) The expression of TNRSF10C among FHC, DLD-1 and HCT-116. (**B**) The expression of TNRSF11A among FHC, DLD-1 and HCT-116. (**C**) The expression of TNRSF13C among FHC, DLD-1 and HCT-116.

**Figure 9:**
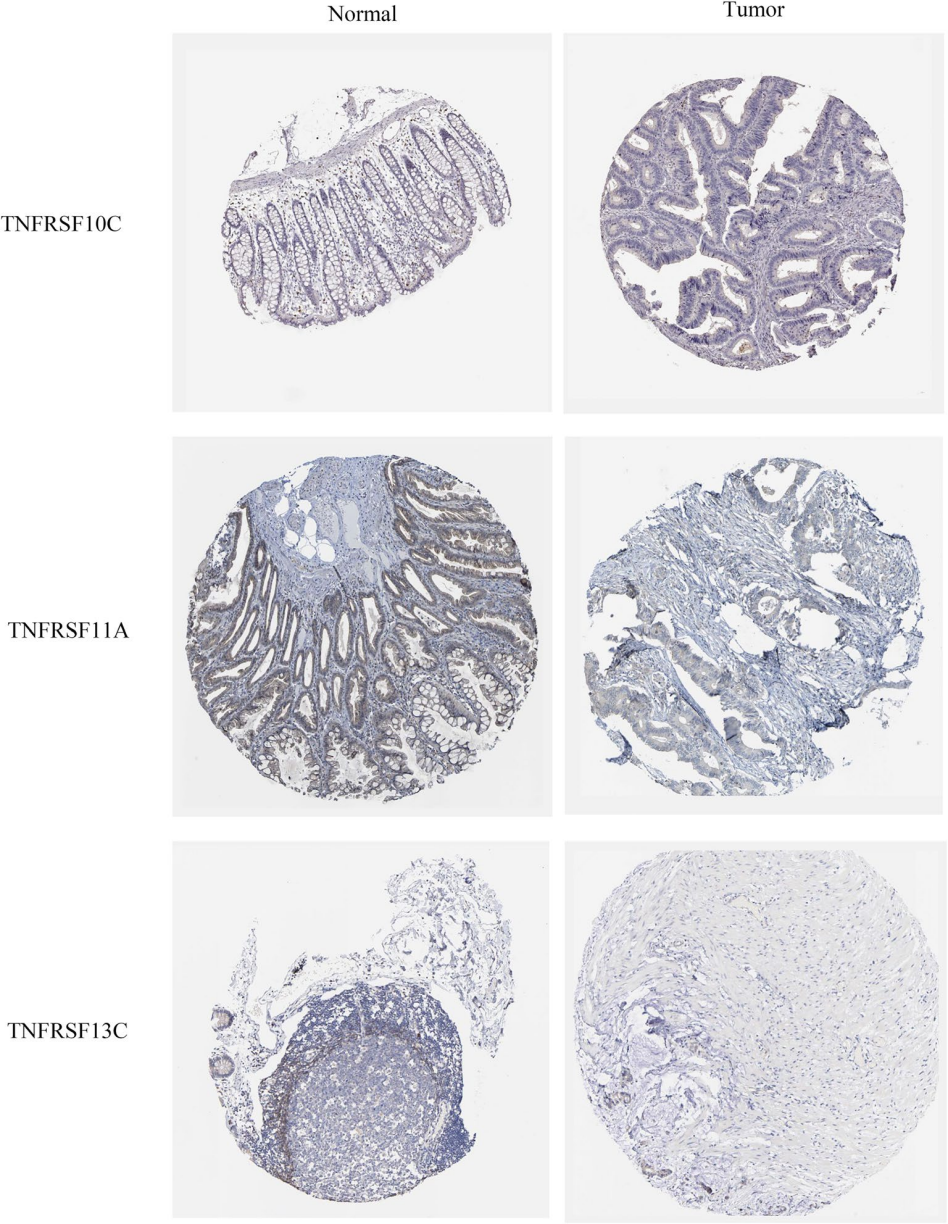
The immunohistochemical analysis of TNRSF10C, TNRSF11A and TNRSF13C were compared between tumor and normal tissues.

Owing to the lack of public data on immune responses in colorectal cancer, we used a melanoma dataset (GSE78220) to validate the risk score. To validate the immune response of the risk models, we compared the ESTIMATE Scores, immune Scores, Stromal Score, Tumor Purity (Fig. 7A), immune checkpoints, HLA (Fig. 7B) and immune receptors in melanoma cohort (GSE78220). It suggests significant differences in immune response between the high-risk and low-risk groups. The results of in vitro experiments are shown in Fig. 8. The expression levels of TNFRSF13C, TNFRSF10C, and TNFRSF11A were statistically significant in FHC and COAD cells, which is consistent with the results of bioinformatic analysis. Subsequently, the results of immunohistochemical analysis of the three genes were compared between tumor and normal tissues using data from a public database (https://www.proteinatlas.org/) (Fig. 9).

### Biological processes and pathways associated with costimulatory molecules

**Figure 10:**
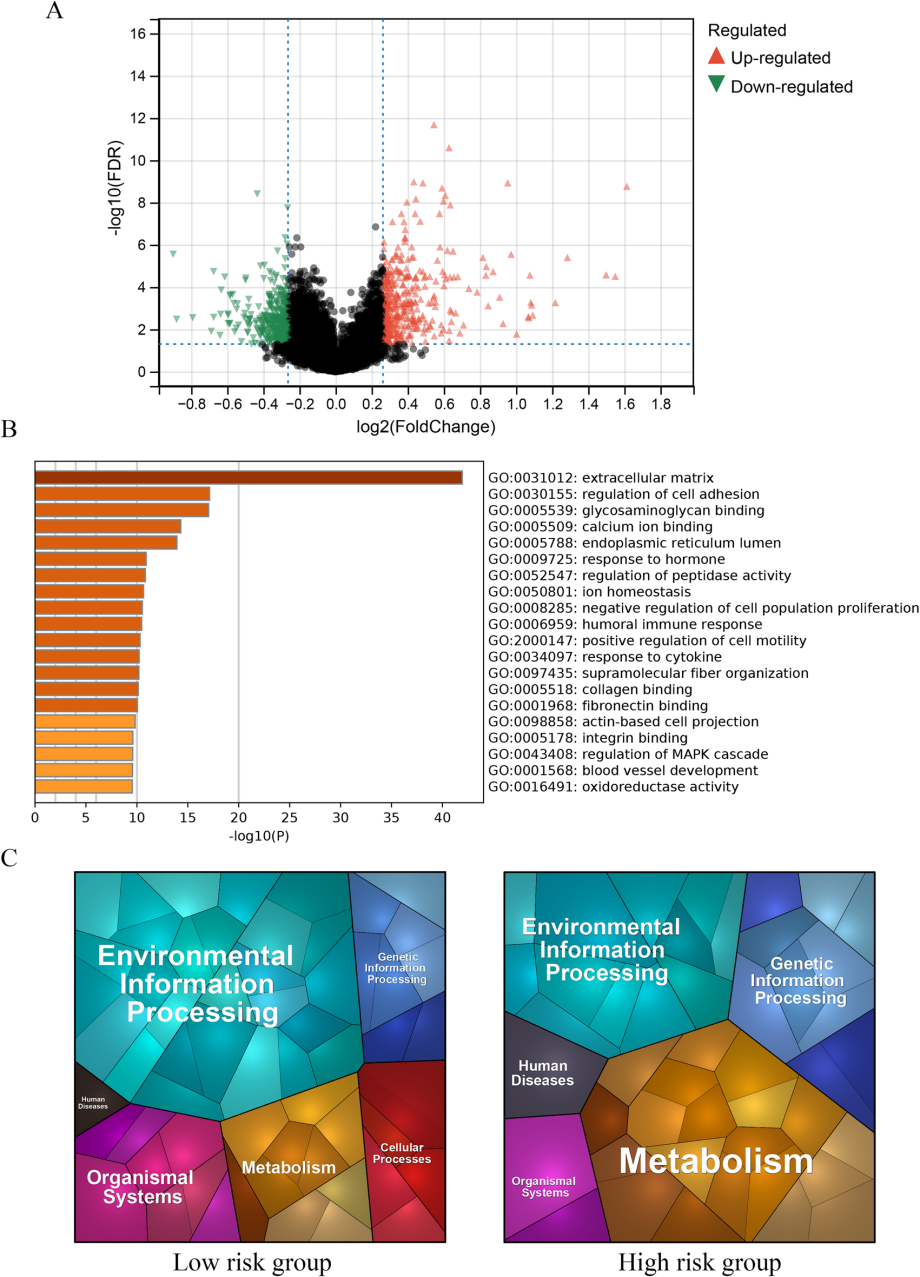
Biological processes and pathways associated with costimulatory molecules (**A**) The volcanic map of mRNAs between the high- and low-risk groups. (**B**) The GO functional analyses in the high- and low-risk groups. (**C**) The KEGG functional analyses in the high- and low-risk groups.

A volcanic map (Fig. 10A) was plotted to demonstrate the differential expression of mRNAs between the high- and low-risk groups. KEGG and GO functional analyses were performed to examine the potential biological mechanisms associated with costimulatory molecules in the high- and low-risk groups (Fig. 10B,C). The top 5 GO terms included extracellular matrix, regulation of cell adhesion, glycosaminoglycan binding, calcium ion binding, and endoplasmic reticulum lumen. The top 5 KEGG terms included environmental information processing, organismal systems, metabolism, cellular processes, and genetic information processing.

### Developing a new nomogram with clinicopathological information

**Figure 11:**
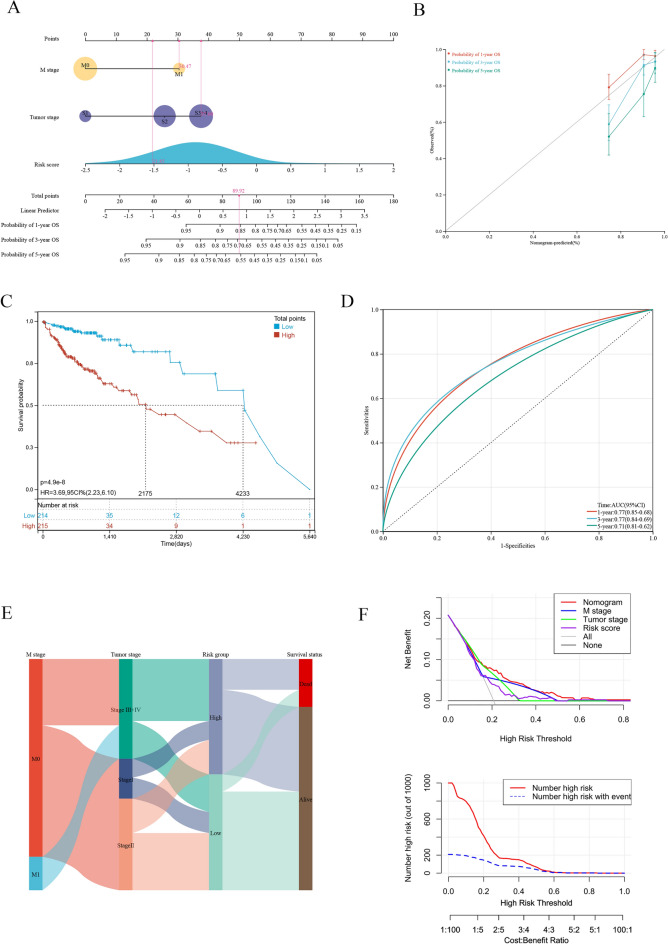
Developing a new nomogram with clinicopathological information (**A**) The nomogram of the risk model. (**B**) The calibration curves of the nomogram of risk model. (**C**) Kaplan–Meier curves for the overall survival of patients in the high- and low-risk groups based on risk model. (**D**) The time-dependent ROC curves supporting prognostic accuracy of the risk score based on risk model. (**E**) Sankey diagram showing the connection degree between the clinicopathological information and survival status. (**F**) The DCA curves of clinical practicability of the nomogram.

To enhance the practicability of the risk model, the clinicopathological data of patients were integrated with the risk scores to construct a nomogram. Multivariate and univariate Cox regression analyses revealed that the M stage, tumor stage, and risk scores were significantly correlated with the prognosis of patients (Table 2). After combining all factors, a nomogram was constructed, and each patient was assigned a score (Fig. 11A). For example, a patient had stage M1 and tumor stage 3–4 COAD. Combined with the risk score, the total score of this patient was 89.92. The nomogram in Fig. 8A demonstrates the 1-, 3-, and 5-year survival rates of patients based on their risk scores and clinical characteristics. A high score indicated a poor prognosis. All influencing factors are shown in Fig. 11E. Calibration curves demonstrated that the nomogram had good accuracy in predicting 1-, 3-, and 5-year OS (Fig. 11B). Additionally, the predictive accuracy of the nomogram was verified in the test set. The 1-, 3-, and 5-year OS of patients is shown in Fig. 11C. The ROC curve of the nomogram is shown in Fig. 11D. The nomogram exhibited good performance in predicting the survival of patients at 1, 3, and 5 years, with AUC values of 0.77, 077, and 0.71, respectively. DCA curves further verified the clinical practicability of the nomogram. Compared with a traditional single clinicopathological feature, the nomogram provided better net benefit (NB) (Fig. 11F).

**Table 2:** Univariate and multivariate analyses of risk factors with OS in the training cohort.

Variables	N	Univariate analysis		Multivariate analysis	
		HR (95% CI)	*P* value	HR (95% CI)	*P* value
Age (years)	429	1.02 (1.00 1.04)	0.091		
Gender					
Female	202	1 (ref)			
Male	227	1.11 (0.73 1.70)	0.627		
T stage					
T1	9	1 (ref)			
T2	75	0.48 (0.05 4.68)	0.531		
T3	297	1.83 (0.25 13.21)	0.551		
T4	48	6.01 (0.80 45.04)	0.081		
N stage					
N0	253	1 (ref)		1 (ref)	
N1	99	1.70 (0.98 2.97)	0.060	0.35 (0.12 1.00)	0.050
N2	77	4.63 (2.82 7.58)	< 0.001	0.76 (0.29 2.04)	0.592
M stage					
M0	367	1 (ref)		1 (ref)	
M1	62	4.65(2.98 7.24)	< 0.001	19.40 (4.62 81.44)	< 0.001
Tumor stage					
Stage I	74	1 (ref)		1 (ref)	
Stage II	170	2.45 (0.73 8.22)	0.146	2.46 (0.73 8.25)	0.146
Stage III + IV	185	7.24 (2.27 23.13)	< 0.001	7.76 (1.67 36.03)	0.009
Risk score	429	2.79(1.79 4.36)	< 0.001	2.12 (1.26 3.56)	0.005

## Discussion

Immunotherapy based on ICIs has revolutionized the treatment of cancer. However, only a small proportion of patients responds to ICIs, and biomarkers that can identify patients who are more likely to respond to immunotherapy are lacking. The basis of ICI therapy is tumor immunogenicity, which is determined by tumor antigenicity and antigen presentation efficiency. At present, the immunotherapeutic drugs successfully used in clinical settings include targeted costimulatory molecules. Targeted therapy with ICIs plays a key role in cancer immunotherapy. It focuses on cytotoxic T lymphocyte antigen 4 (CTLA4) and PD1 receptors, which are members of the CD28 family. The effective killing of tumor cells by the immune system greatly depends on the induction of tumor-specific T-cell responses and inhibition of tumor growth, which are also the theoretical basis of immunotherapy^23^. Antigens in the human immune system cannot sufficiently drive the activation of naive T cells. Activation of T cells requires signals from two sources^24^. Both signals are transmitted by antigen-presenting cells (APCs) to inactive T cells. The first signal depends on the specific recognition of antigens by the T cell receptor (TCR)/CD3 complex after treatment of APCs. The second signal is transmitted by the interaction between costimulatory molecules on APCs and the surface of T cell-related receptors. Importantly, costimulatory molecules are vital for the proliferation, differentiation, and survival of T cells to maintain normal immune function. However, several costimulatory molecules can constrain or activate T cell function when antigens are continuously expressed and stimulated; therefore, they can be used as immunotherapeutic targets. At present, drugs targeting costimulatory molecules, such as PD1 and CTLA-4 inhibitors, are successfully used in clinical settings^25^. Therefore, further study of costimulatory molecules can help to make better use of the immune system to eliminate cancer cells and predict the response of patients to immunotherapy.

This study suggests that the risk model developed based on costimulatory molecules is an effective tool to predict the prognosis and immunotherapy response of patients with COAD. Higher risk scores and more advanced clinical stages indicated a poor prognosis. Therefore, we combined the traditional clinicopathological features and risk scores to develop an exclusive prognosis evaluation system. Additionally, we established a nomogram to demonstrate the relationship between costimulatory molecules and clinical prognosis. The findings of this study provide novel insights into the development of risk stratification and immunotherapeutic strategies.

The differential expression of 59 costimulatory molecules was analyzed in COAD and normal tissues. Univariate and multivariate Cox regression and Lasso regression analyses were performed to identify three costimulatory molecules related to the prognosis of COAD, namely, TNFRSF10C, TNFRSF11A, and TNFRSF13C. Although various machine learning methods can be used to select the right variables, determining the best method remains a problem. Many previous studies have reported the comparison between Lasso and other machine learning methods (ridge and elastic net regression) and have identified Lasso regression as the most suitable machine learning method. Therefore, we used Lasso regression in this study^26,27^. The three costimulatory molecules identified in this study are members of the TNF superfamily. These findings indicate that the TNF superfamily plays a greater role in the prognosis and immunotherapeutic response of patients with COAD. TNFRSF10C is one of the most common missing gene loci in patients with CRC^28^.

TNFRSF10C, also named decoy receptor-1 (DcR1) and TRAIL-R3, acts as one of the TRAIL decoy receptors and can inhibit the signaling pathway of intracellular apoptosis to protect cells from TRAIL-induced apoptosis^29^. The expression of TNFRSF10C is often downregulated in tumor tissues, and a reduction in its copy number can promote distant metastasis in CRC^30^. In addition, the TRAIL gene can predict the treatment response and prognosis of patients with CRC, glioblastoma, and breast cancer^31–36^. TNFRSF11A, also known as NF-κB receptor activator (RANK), can activate various signaling pathways, such as NF-κB, JNK, ERK, p38α, and Akt/PKB^37^. TNFRSF11A signaling can promote cell proliferation and inhibit apoptosis^38^. However, several studies have shown that RANK/TNFRSF11A may promote apoptosis and inhibit cell proliferation^39^. TNFRSF13C, a BAFF receptor (BAFFR), is a key regulator of the proliferation, development, and maturation of B cells^40^. It is affiliated with drug-resistant B cells and affects the prognosis and immunotherapy response of patients with lung adenocarcinoma^41^.

The risk model developed in TCGA-COAD cohort was verified in an external dataset. Survival analysis and ROC curves revealed that the prognosis of patients gradually deteriorated with an increase in risk scores. The results of validation analysis were consistent with those obtained in the training dataset. To further improve the accuracy of the risk model, the risk score was integrated with traditional clinicopathological features. Higher risk scores indicated advanced N, M, and tumor stages. Subsequently, a nomogram integrating clinicopathological features and risk scores was established. The AUC values of the final model for predicting 1-, 3-, and 5-year survival were 0.77, 0.77, and 0.71, respectively, which were significantly better than those of the risk model. Finally, the clinical decision-making curve demonstrated that the risk model was better than the traditional predictive model.

The immunotherapy response was better in the low-risk group, suggesting that immunotherapeutic efficacy was better in patients with low risk scores than in those with high risk scores. These findings suggest that the ability of the autoimmune system to clear tumor cells is poor in patients with high risk scores ^13^. Additionally, the low-risk group had higher stromal, immune, and ESTIMATE scores, which is consistent with the abovementioned results. Analysis of HLAs revealed the presence of several high-expression sites in the high-risk group, which may be used as new immunotherapeutic targets in the future.

In this study, we constructed a risk signature based on costimulatory molecules to predict the prognosis of COAD and stratified patients based on risk scores to guide immunotherapy and improve prognosis. To the best of our knowledge, this study is the first to report a risk model integrated with prognostic costimulatory molecules and clinicopathological features for predicting the prognosis of COAD. The findings of this study may help clinicians to improve the evaluation of the prognosis of COAD and develop immunotherapeutic strategies according to the signature. However, this study has some limitations. First, this study had a retrospective design and was based on public databases. This study was entirely performed using bioinformatic methods. Therefore, prospective studies are required to verify the predictive ability of the risk model. We speculate that neo-adjuvant chemotherapy, radiation therapy, or adjuvant chemotherapy can affect the validity of the model. However, the currently available public databases do not contain relevant information. In the future, we will not only consider the impact of neo-adjuvant chemotherapy, radiation therapy, and adjuvant chemotherapy on the risk model but also extract more local data to predict immunotherapy responses and perform in vivo and in vitro experiments to validate and refine the risk model.

## Conclusion

We established a risk model based on the costimulatory molecules TNFRSF10C, TNFRSF11A, and TNFRSF13C to predict the prognosis of COAD. The immunotherapy response of patients with COAD can be predicted using this model. Overall, the risk model represents a novel prognostic biomarker for COAD.

## Supplementary Information

Supplementary Information 1.

Supplementary Information 2.

Supplementary Information 3.

Supplementary Information 4.

## Data Availability

TCGA gene expression profiles and patients’ clinical data in this study are available at UCSC Xena (https://xena.ucsc.edu/). Gene mutation data could be acquired from TCGA data portal (https://portal.gdc.cancer.gov/). The datasets used and/or analyzed during the current study are available from the corresponding author on reasonable request.

## Abbreviations

COAD: Colon adenocarcinoma
TCGA: The cancer genome atlas
TNF: Tumor necrosis factor
TNFSF: TNF ligand superfamily
Lasso: Least absolute shrinkage and selection operator
ROC: Receiver operating curve
ICB: Immune checkpoint blockade
KEGG: Kyoto encyclopedia of genes and genomes
GO: Gene ontology
DCA: Decision curve analysis
HLA: Human leukocyte antigen
PPI: Protein–protein interaction
GEO: Gene expression omnibus data base
HPA: Human protein atlas
