# Supplementary figures and images for: Prognostic costimulatory molecule-related signature risk model correlates with immunotherapy response in colon cancer
Source: Sci Rep. 2023 Jan 16;13:789. doi: 10.1038/s41598-023-27826-7 (PMC9842650; doi:10.1038/s41598-023-27826-7)

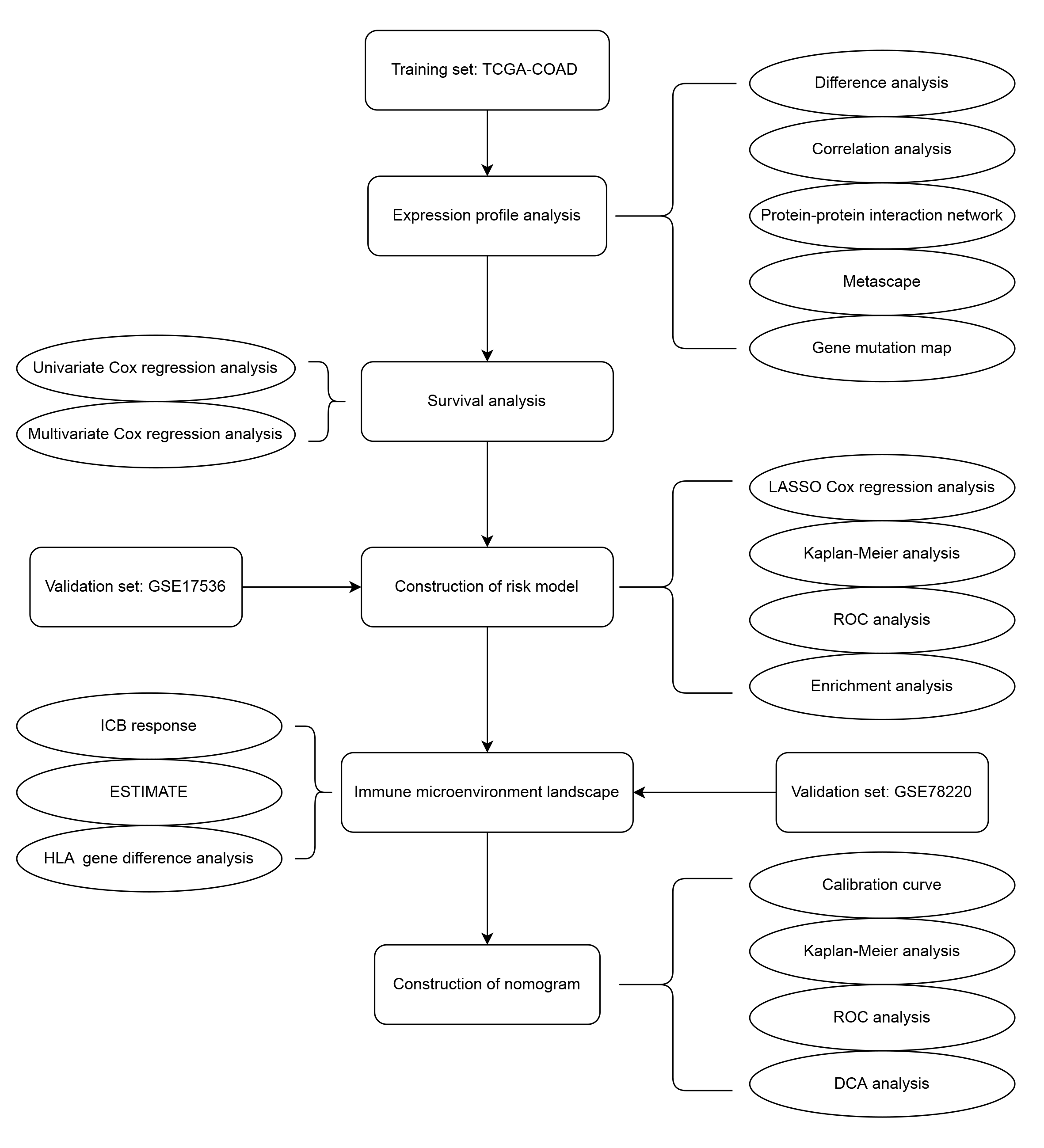

Supplement: Supplementary file 1 — Supplementary Information 1. [file 41598_2023_27826_MOESM1_ESM.tif]

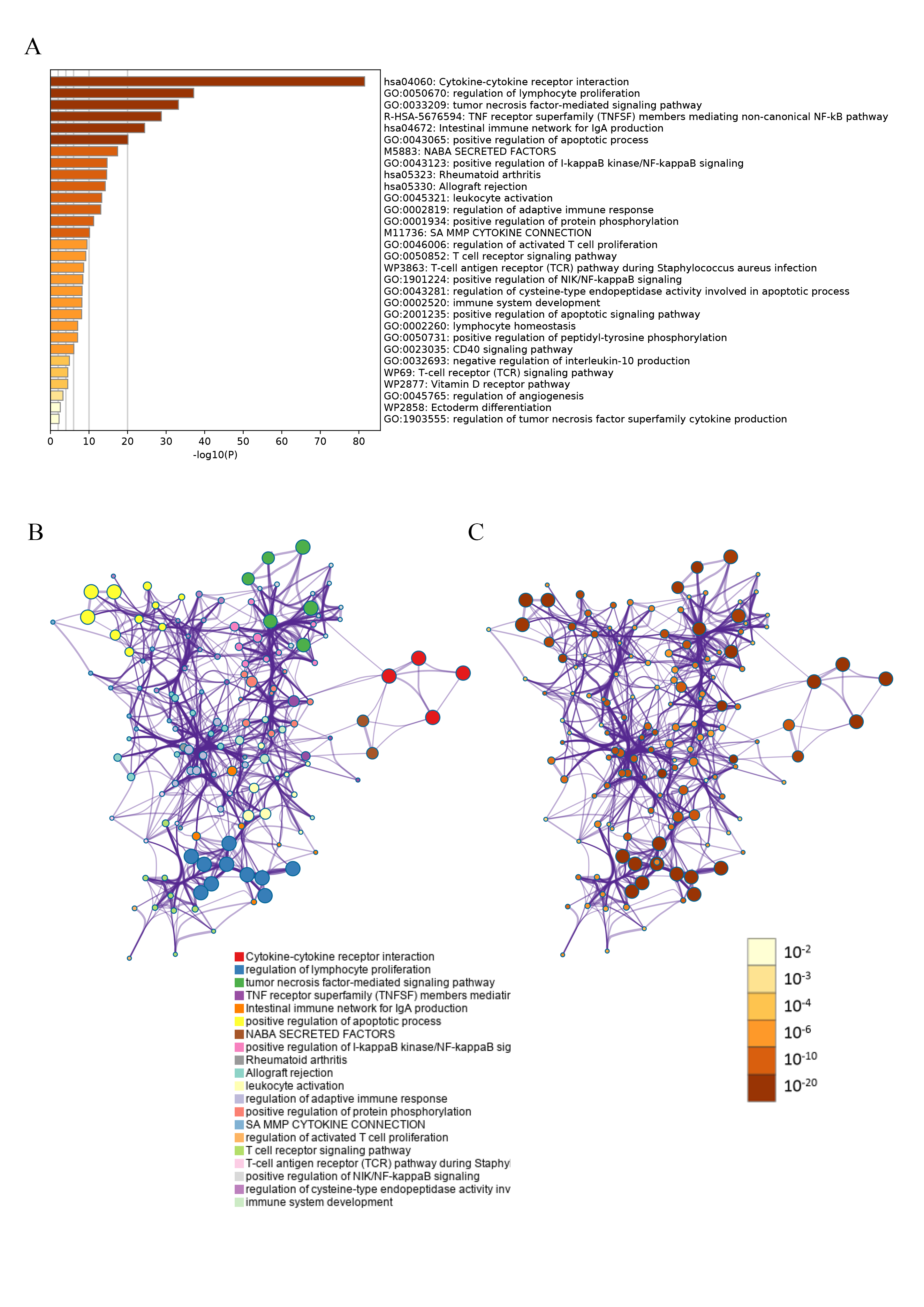

Supplement: Supplementary file 2 — Supplementary Information 2. [file 41598_2023_27826_MOESM2_ESM.tif]
