# Supplementary material for: Prognostic costimulatory molecule-related signature risk model correlates with immunotherapy response in colon cancer
Source: Sci Rep. 2023 Jan 16;13:789. doi: 10.1038/s41598-023-27826-7 (PMC9842650; doi:10.1038/s41598-023-27826-7)
Supplement: Supplementary file 4 — Supplementary Information 4. [file 41598_2023_27826_MOESM4_ESM.docx]

**Supplement Table S2**. The sequence of primers involved in this study.

| Gene | Sequence (5’-3’) | |
| --- | --- | --- |
| beta-actin | Forward | CATGTACGTTGCTATCCAGGC |
|  | Reverse | CTCCTTAATGTCACGCACGAT |
| TNFRSF11A | Forward | TGCTGTCACTTCTGCCACATTCTG |
|  | Reverse | CTACCCTCTCATTCCGCAACTCTTC |
| TNFRSF10C | Forward | CCCTAAAGTTCGTCGTCGTCATCG |
|  | Reverse | GCTGTGCCTCTGTTGCTGTGG |
| TNFRSF13C | Forward | TCACTGTAACCTCCGACTCCTTGG |
|  | Reverse | GGTGGTGCGTGCCTGTAATCC |

RT, reverse transcription.
